# On the Use of Iodide of Potassium in Asthma

**Published:** 1845-03

**Authors:** W. B. Casey

**Affiliations:** Middletown, Connecticut


*On the Use of Iodide of Potassium in Asthma.* By W. B. Casey, M.D., of Middletown, Connecticut. Mr. Editor: For a year past I have looked through the various journals that came under my observation, in the expectation of seeing some notice of the subject of this article; but have, up to this time, met with but a mere incidental mention of the fact that hydriodate of potash has been employed with some benefit in asthma. I therefore send you the result of my experience with it, trusting that the statement which I make will excite the attention of our profession, and lead to a trial of the remedy on a more extensive scale. Some two years since, Professor Mütter, of Philadelphia, mentioned in the course of conversation with me, that he had accidentally discovered the fact to which I am now endeavoring to draw attention.

I immediately after this commenced its use with a number of patients suffering under this distressing and intractable complaint, and to our mutual delight, they were all more or less speedily relieved. I have now made use of the medicine in some twenty-five or thirty cases of asthma, some of them very severe and aggravated; and so far, in no one instance where a fair trial has been made, has it failed to afford unequivocal and decided relief. These facts I have stated at our county medical meetings, and have urged the adoption of the practice upon many of our neighboring physicians; and so far as I can ascertain the result, all who have tried the remedy will endorse my statement of its value. I am happy to add the testimony of Dr. North, an eminent physician resident at Saratoga Springs, who informs me that he has experienced in his own person, great relief from the use of the medicine, and has witnessed the same effect in others. As a general rule, the patient is benefitted after a few days' employment of the article, but some cases will require more time, perhaps weeks before they improve; in one of mine, a very severe case of over twenty years' duration, I persevered for nearly three months before there was any decided amendment.

I feared, at the outset, that the medicine would prove to be merely a palliative, (and even then it would be invaluable,) but further experience warrants my belief that in mild cases of recent date a cure may be effected by its means.

In almost one-fourth of my cases, relapses have occurred after discontinuing the remedy; this occurrence, however, was, in most of them, owing to severe attacks of catarrh, or to errors in diet and consequent derangement of the digestive organs, which, by the way, should never be overlooked in the treatment of asthma; I may mention in this connection, that most of my patients, while using this medicine, had an excellent appetite and gained flesh rapidly.

A long continued use of the iodide of potassium will, in some subjects, occasion an eruption, generally of a pustular form, (almost always ecthyma;) and I have twice been disposed to attribute to it the occurrence of a slight conjunctivitis; the omission of the medicine for a few days, together with a few doses of rhubarb and soda, will be found sufficient for the removal of these inconveniences. It would scarcely be worth while to offer at the present time any explanation of the modus operandi of this medicine in asthma; nor is it necessary here to describe the symptoms, pathology, &c., of the disease, my sole object being to bring into notice what I have found a successful remedy for a distressing complaint. Extended experience must determine its value and applicability.

From two to five grains of the iodide of potassium, given three times a day, dissolved in water or some syrup, as, for instance, that of sarsaparilla or tolu, will generally be found sufficient for ordinary cases of the disease. Its continuance must be regulated by the circumstances of each case; of course no intelligent practitioner need be reminded of the attention requisite as regards diet, clothing, and exercise. Dr. North stated that he had taken ʒss. at a dose at bedtime, without inconvenience, and with the effect of preventing the paroxysm usually occurring at night.

Some of your readers may perhaps find fault with this paper, on account of its crudeness of style, and want of method and arrangement; but the practical physician, who will make fair trial of the remedy, will hardly be disposed to criticise the style of the article, or consider as wasted the time spent in its perusal.

*Note.*—It may not be uninteresting or irrelevant to mention that I have given the hydriodate of potash to several horses troubled with "the heaves or hives," (I am doubtful as to its orthography,) and in all, while under the influence of the medicine, the disease was suspended.

*New York Journal of Medicine.*

